# Promiscuous plasmid replication in thermophiles: Use of a novel hyperthermophilic replicon for genetic manipulation of *Clostridium thermocellum* at its optimum growth temperature

**DOI:** 10.1016/j.meteno.2016.01.004

**Published:** 2016-01-29

**Authors:** Joseph Groom, Daehwan Chung, Daniel G. Olson, Lee R. Lynd, Adam M. Guss, Janet Westpheling

**Affiliations:** a Department of Genetics, University of Georgia, Athens, GA, USA; b The BioEnergy Science Center, Oak Ridge National Laboratory, Oak Ridge, TN, USA; c National Renewable Energy Laboratory, Biosciences Center, Golden, CO, USA; d Thayer School of Engineering at Dartmouth College, Hanover, NH, USA; e Biosciences Division, Oak Ridge National Laboratory, Oak Ridge, TN, USA

**Keywords:** 5-FOA, 5-fluoroorotic acid, FUdR, 5-fluoro-2′-deoxyuridine, AZH, 8-azahypoxanthine, PCN, plasmid copy number, Ct, cycles to threshold., Plasmid, Genetic tools, Transformation, Thermophile, Clostridia

## Abstract

*Clostridium thermocellum* is a leading candidate for the consolidated bioprocessing of lignocellulosic biomass for the production of fuels and chemicals. A limitation to the engineering of this strain is the availability of stable replicating plasmid vectors for homologous and heterologous expression of genes that provide improved and/or novel pathways for fuel production. Current vectors relay on replicons from mesophilic bacteria and are not stable at the optimum growth temperature of *C. thermocellum*. To develop more thermostable genetic tools for *C. thermocellum*, we constructed vectors based on the hyperthermophilic *Caldicellulosiruptor bescii* replicon pBAS2. Autonomously replicating shuttle vectors based on pBAS2 reproducibly transformed *C. thermocellum* at 60 °C and were maintained in multiple copy. Promoters, selectable markers and plasmid replication proteins from *C. bescii* were functional in *C. thermocellum*. Phylogenetic analyses of the proteins contained on pBAS2 revealed that the replication initiation protein RepL is unique among thermophiles. These results suggest that pBAS2 may be a broadly useful replicon for other thermophilic Firmicutes.

## Highlights

• Intergeneric replication of a hyperthermophilic plasmid replicon in *C. thermocellum.*
• Transformation efficiencies >3000 colony forming units per µg of plasmid DNA.
• Plasmid maintained at ~20 copies per chromosome in *C. thermocellum*.
• *Caldicellulosiruptor* proteins and promoters function in *C. thermocellum.*
• The pBAS2 RepL replication initiation protein is unique among thermophilic proteins.

## 1 Introduction

*Clostridium thermocellum* has been the subject of intense study because of its unique mechanism for solubilizing plant biomass. *C. thermocellum* secretes proteins that form an extracellular organelle, called a cellulosome (Bayer et al., 2013, Lamed and Bayer, 1988), for attachment to and digestion of complex plant biomass. Its ability to produce ethanol from cellulose has made it a leading candidate for consolidated bioprocessing (Akinosho et al., 2014, Demain et al., 2005). Recent work to engineer *C. thermocellum* for increased ethanol production has eliminated competing fermentation pathways (Argyros et al., 2011, Biswas et al., 2015, Deng et al., 2013, Papanek et al., 2015, Tripathi et al., 2010) and allowed synthesis of other fuel molecules like isobutanol (Lin et al., 2015).

Published genetic methods in *C. thermocellum* allow transformation of plasmid DNA (Guss et al., 2012, Olson and Lynd, 2012a, Tyurin et al., 2004), the generation of gene deletions (Argyros et al., 2011) and some methods for gene expression (Deng et al., 2013, Lin et al., 2015, Mearls et al., 2015, Olson et al., 2015). While gene deletions and gene expression have led to significant increases in ethanol production, the expression systems for robust high-level expression of homologous and heterologous proteins in *C. thermocellum* have limitations. The plasmid replicon from pNW33N that has been used extensively in published *C. thermocellum* transformation protocols (Tripathi et al., 2010) is not stable at elevated temperatures (Olson and Lynd, 2012b). Therefore, current methods for DNA transformation require that selection of transformants be performed at 50–51 °C (Olson and Lynd, 2012a), a suboptimal growth temperature for *C. thermocellum*, which grows optimally at 60 °C (McBee, 1954). Because this origin does not support stable replication at 60 °C, it may contribute to both the observed plasmid instability (Olson et al., 2015) and chromosomal integration (Lin et al., 2015), even at 50–55 °C. A plasmid that could faithfully replicate at 60 °C would enable optimal *in vivo* expression and rapid screening of cell wall decomposition enzymes or metabolic enzymes for strain engineering.

Selectable markers that have been used in *C. thermocellum* (Olson and Lynd, 2012a) include *pyrF*, that confers uracil prototrophy in a *pyrF* deletion strain; chloramphenicol acetyl transferase (*cat*), that confers thiamphenicol resistance; and neomycin acetyl transferase (*neo*), that confers neomycin resistance. Counter-selectable markers include *pyrF*, that confers sensitivity to 5-fluoroorotic acid (5-FOA); *tdk*, that confers sensitivity to 5-fluoro-2′-deoxyuridine (FUdR); and *hpt*, that confers sensitivity to toxic purine analogs such as 8-azahypoxanthine (8AZH) (Pritchett et al., 2004, Stout and Caskey, 1985). Counter-selection with *tdk* may be performed in a wild type strain, while use of *pyrF* and *hpt* require the use of a strain deleted for the *C. thermocellum* chromosomal copy of the gene.

Many plasmids have been shown to be capable of intergeneric and even interphyletic replication, raising the possibility of finding additional origins of replication for use in *C. thermocellum*. Early work in mesophilic Gram-positive bacteria identified several such replicons. *Staphylococcus aureus* plasmid pC194, that replicates by a rolling circle mechanism (Khan, 2005), as well as other *Staphylococcus* plasmids successfully transformed *Bacillus subtilis* (Dubnau, 1983, Ehrlich, 1977). *Enterococcus faecalis* plasmid pAMβ1 was used to transform *Lactobacillus acidophilius* (Luchansky et al., 1988) and *B*. *subtilis* (Jannière et al., 1990), and has since become a broad-range host vector for members of the Firmicutes. An example of interphyletic plasmid replication is pNG2, that was isolated from *Corynebacterium diphtheriae*, a member of the Actinobacteria phylum, but replicates in *Escherichia coli*, a member of the Proteobacteria phylum (Serwold-Davis et al., 1987).

Unfortunately, many of the commonly used plasmid origins were isolated from mesophilic organisms, so functionality at thermophilic temperatures is a concern. For instance, most Staphylococcal plasmids can be cured by growth at 43 °C (McNamara, 2008). The *B. subtilis* plasmid pIM13 was used to transform *S*. *aureus* (Projan et al., 1987), and more recently the replicon was used for the construction of pIKM1 for use in the thermophile *Thermoanaerobacterium saccharolyticum* at 48 °C*,* although its optimal growth temperature is 60 °C (Mai et al., 1997). The pIP404 replicon (Garnier and Cole, 1988) from *Clostridium perfringens,* that grows optimally at 43 °C, was used to transform *Thermoanaerobacter ethanolicus* JW200*,* albeit at a temperature much lower than the recipient's optimum growth temperature of 69 °C (Peng et al., 2006). Although the promiscuity of many plasmids has been demonstrated, there are limitations to mesophilic bacterial plasmids being used at the optimal growth temperatures of thermophiles. Issues of structural instability (Olson et al., 2015) chromosomal integration (Lin et al., 2015, Olson et al., 2015), and decreased copy number (Klapatch et al., 1996) have been reported.

An alternative approach is to identify origins of replication from thermophilic organisms as a starting point for developing more thermostable shuttle vectors. One example is plasmid pBAS2 that is native to *Caldicellulosiruptor bescii* (Clausen et al., 2004), a thermophilic Firmicute. *C. bescii* grows optimally at 75 °C (Yang et al., 2009) and plasmids using the pBAS2 origin of replication have been shown to replicate at high copy at 65 °C (Groom et al., 2014). The replication functions of pBAS2 have been used to construct vectors for transformation of *C. bescii* (Chung et al., 2013, Chung et al., 2015) and *Caldicellulosiruptor hydrothermalis* (Groom et al., 2014). Bioinformatic analysis revealed significant sequence identity to other Gram-positive rolling circle replication (RCR) origins, but the mechanism of pBAS2 replication is not clear. The conserved nick site is not obvious, and single stranded DNA that typically accumulates as a consequence of rolling circle replication was not observed for pBAS2 (Clausen et al., 2004). The pBAS2 replicon encodes a replication protein with homology to known RepL proteins and a Xer-like recombinase (Chung et al., 2013, Clausen et al., 2004), which is potentially responsible for the resolution of multimers of both chromosomes and plasmids that form during replication (Colloms, 2013, Subramanya et al., 1997). The open reading frame encoding Cbes2777 (the Xer-like recombinase XerD) was shown to be necessary for autonomous replication in *C. bescii* (Chung et al., 2013). While some replicons rely on host recombinases that work *in trans* to resolve multimers, pBAS2 encodes its own recombinase to resolve plasmid multimers and maintain sequence fidelity.

Based on the thermophilic source of pBAS2 and the presence of both a replication gene and a recombinase gene, we hypothesized that pBAS2-derived plasmids would be capable of independent replication in *C. thermocellum* at its optimal growth temperature of 60 °C. We therefore built and tested plasmids carrying the pBAS2 origin of replication with different selectable markers to explore this possibility. Here we report the transformation and stable replication in *C. thermocellum* of plasmid vectors based on pBAS2. We show that the *pyrF* gene from *C. bescii* functions to complement a deletion of the *pyrF* gene in *C. thermocellum* and that promoters from *C. bescii* function to drive expression of genes at levels sufficient for selection of transformants. Transformation of *C. thermocellum* DSM 1313 was performed at 60 °C, the optimal growth temperature of this strain, and plasmids derived from pBAS2 replicate stably in *C. thermocellum* at this temperature. Phylogenetic analysis of pBAS2 protein sequences suggests that the replication protein is novel and unique among known thermophilic proteins.

## 2 Methods

### 2.1 Bacterial media and growth conditions

*C*. *thermocellum* DSM 1313 and its derivatives were grown anaerobically in modified CTFUD medium (Olson and Lynd, 2012a) at 60 °C, under an atmosphere of 85% nitrogen, 10% CO_2_, and 5% Hydrogen. Defined medium for transformation and selection was CTFUD-NY (Olson and Lynd, 2012a), which contains a vitamin mix of p-aminobenzoic acid, vitamin B12, biotin, and pyridoxamine in place of the yeast extract. CTFUD-NY was supplemented with 360 µM uracil when noted. Complex medium for recovery after transformation was similar to CTFUD but contained casein (0.2% w/v) and less yeast extract (0.1% w/v), which is referred to as CTFUD+C. *E*. *coli* was grown in Luria-Bertani broth supplemented with 50 µg/mL apramycin when selecting for the presence of a plasmid.

### 2.2 Plasmid vector construction

All PCR reactions for cloning were performed with Q5 polymerase (New England Biolabs, Ipswich, MA) according to the manufacturer's instructions (98 °C duplex denaturation, 60 °C annealing temperature, 30 s per kb at 72 °C for elongation). Plasmid pDCW196 was constructed by ligating *C. bescii* shuttle vector pDCW89 (Chung et al., 2013) to the *cat* gene from *C. thermocellum* vector pMU612 (Tripathi et al., 2010). Primers X013 and X014 amplified a 7.69 kb fragment from pDCW89, and primers X015 and X016 amplified a 1.053 kb fragment from pMU612. These fragments were digested with *Bam*HI and *Nde*I (New England Biolabs) twice in succession for 45 min at 37 °C and ligated with the FastLite ligation kit (Epicentre, Madison, WI) according to the manufacturer’s instructions. Plasmid pJGW37 was constructed by amplifying a 7.467 kb fragment from pDCW196 using primers JG024 and JG099, digesting with *Xba*I (New England Biolabs) and ligating as described above to circularize the linear fragment. All plasmid sequences were verified by Sanger DNA sequencing (Genewiz). All primers are listed in Supplementary Table S1.

### 2.3 Transformation of *C. thermocellum* Δ*pyrF*

A 20 mL starter culture of *C. thermocellum* DSM 1313 Δ*pyrF* was grown at 60 °C to mid-exponential phase in defined CTFUD medium (Olson and Lynd, 2012a) supplemented with uracil. 15 mL of this culture was transferred to 150 mL of the same medium and grown to OD_600_=0.6. Cells were cooled to room temperature for 25 min, harvested aerobically at 7500×*g* for 10 min, and washed twice with pre-chilled 10% sucrose. Competent cells were divided into 30 µL aliquots, and those not used immediately were frozen in a dry ice–ethanol slurry and stored at −80 °C. 500 ng of plasmid DNA pDCW89 was incubated with each aliquot for 15 min in pre-chilled 1 mm cuvettes. Cells were electrotransformed with a single exponential pulse with a Gene Pulser (BioRad, Hercules, CA) set at 1.8 kV, 25 μF, and 350 Ω, and placed immediately into 60 °C CTFUD+C medium (Olson and Lynd, 2012a) supplemented with 360 µM uracil for recovery. A 0.25% inoculum was transferred to defined liquid CTFUD-NY medium lacking uracil every three hours over the period of recovery to select for transformants.

### 2.4 Transformation of *C.thermocellum* Δ*hpt*

A 10 mL starter culture of *C. thermocellum Δhpt* was grown at 60 °C to mid-exponential phase in rich CTFUD medium (Olson and Lynd, 2012a). This culture was transferred to 150 mL of the same medium and grown to OD_600_=0.9. Cells were cooled to room temperature for 25 min, harvested aerobically at 7500×*g* for 8 min, and washed twice with pre-chilled 10% glycerol, 260 mM sucrose. Competent cells were divided into 30 µL aliquots, and those not used immediately were frozen in a dry ice–ethanol slurry and stored at −80 °C. 350 ng of plasmid DNA pJGW37 was incubated with each aliquot for 15 min in pre-chilled 1 mm cuvettes. Cells were electrotransformed with a single exponential pulse with a BioRad Gene Pulser (1.8 kV, 25 μF, 350 Ω), and placed immediately into warm CTFUD+C medium for 6 h at 60 °C, after which they were serially diluted onto CTFUD plates containing yeast extract (0.1% w/v) and 10 µg/mL thiamphenicol (Sigma, St. Louis, MO). Colonies appeared after 4–5 days of growth at 60 °C under an atmosphere of 85% nitrogen, 10% CO_2_, and 5% Hydrogen. Transformation efficiency was calculated in CFU/µg of plasmid DNA, and experiments were performed in biological triplicate.

### 2.5 Total DNA isolation from *Clostridium thermocellum*

For plasmid copy number determination over the growth phase, a 0.1% (v/v) inoculum was grown without shaking at 60 °C. 2% (v/v) samples were removed for qPCR analysis at the indicated time points (Fig. 3). For plasmid maintenance experiments (Table 2), 0.25% inocula were grown in serial passages to exponential phase (OD_600_~0.4 for *C. thermocellum* Δ*pyrF*, ~0.7 for *C. thermocellum* Δ*hpt*) and 5 mL of culture was harvested at 4 °C at 6100×*g*. The pellet was resuspended in 200 µL 40 mM EDTA, 50 mM Tris–HCl, 25% sucrose (w/v) with 30 mg/mL lysozyme (Sigma, St. Louis, MO) and 1 µL/mL RNase A (Qiagen), and incubated for 30 min at 37 °C. Cells were frozen and thawed five times using a dry ice–ethanol slurry and a 37 °C water bath. 500 µL 6 M Guanidine-HCl pH 8.5 (Sigma, St. Louis, MO) was immediately added to the cell lysate and allowed to incubate at 75 °C for 10 min. The lysate was washed twice with Phenol:chloroform:isoamyl alcohol and once with chloroform. To the aqueous layer, 3 M sodium acetate pH 5.2 was added, and DNA was precipitated at −80 °C for 3 h, washed with cold 70% ethanol and resuspended in 10 mM Tris–HCl. Total DNA for *C. thermocellum* transformants containing pJGW37 was visualized on a 1% agarose gel (Sigma, St. Louis, MO) stained with ethidium bromide (Fig. 2C).

### 2.6 Verification of plasmid transformation and structural stability

Taq polymerase (Sigma, St. Louis, MO) was used for PCR reactions to confirm presence of the plasmid using total DNA purified from *C. thermocellum* transformants as template. Reactions were performed with primers DC091 and DC508 according to the manufacturer's instructions (94 °C duplex denaturation, 56 °C annealing temperature, 1 min per kb at 72 °C for elongation). PCR products were visualized on a 1% agarose gel with an NEB 1 kb Ladder for size verification (NEB, Ipswich, MA). To verify structural stability in *C. thermocellum,* total DNA was electrotransformed into *E. coli* BL21 with a BioRad Gene Pulser (Biorad, Hercules, CA) using an exponential pulse in a 2mm cuvette (2.5 kV, 25 μF, 200 Ω). Selection in *E. coli* was performed with 50 µg/mL apramycin, and colonies were screened for the presence of the plasmid by performing restriction digests with EcoRI and ApaLI (NEB, Ipswich, MA).

### 2.7 Quantitiative polymerase chain reaction (qPCR)

qPCR experiments were carried out with a LightCycler 480 Real-Time PCR instrument (Roche, Basel, Switzerland) with LightCycler 480 SYBR Green I master mix (Roche). Cycle thresholds resulting from amplification with two independent sets of primers specific to either the pJGW07 plasmid (Q1/Q2 inside Cbes2777, Q3/Q4 inside Cbes2778) or the *C. thermocellum* chromosome (CTQ1/CTQ2 inside Clo1313_0092, CTQ3/CTQ4 inside Clo1313_0090) were used to compute relative copy number of the plasmid to the chromosome. The formula for this calculation was PCN=2^| Ct_chromosome_−Ct_plasmid_ |. Four replicate reactions for each primer set were performed, and the average of the two primer sets on each sample was used to calculate the plasmid copy number in each serial subculture (Table S1) according to the method of Lee et al. (2006). Amplification efficiency over 10^4^ fold range of DNA concentration was 102%, within the ratio of 90–110% that is considered acceptable.

### 2.8 Bioinformatic analysis

The National Center for Biotechnology Information (NCBI) and the Kyoto Encyclopedia of Genes and Genomes (KEGG) were used to search for homologous proteins to the ORFs on plasmid pBAS2. Amino acid similarity was also determined with the NCBI Basic Local Alignment Search Tool (BLAST). Clustal Omega was used for multiple sequence alignment with default settings (Goujon et al., 2010, Sievers et al., 2011). ProtTest 3.4 (Darriba et al., 2011) was used to estimate models of evolution for the multiple sequence alignments. To determine posterior probabilities, MRBAYES version 3.2.5 ×64 (Huelsenbeck and Ronquist, 2001) was run for 1,000,000 generations with the WAG+I+G+F model of evolution (Whelan and Goldman, 2001) for Cbes2777 and the LG+I+G+F model (Le and Gascuel, 2008) for Cbes2780. Figtree version 1.4.2 (http://tree.bio.ed.ac.uk/ software/figtree/) was used to visualize the phylogenetic trees generated by MRBAYES. MEME was used for motif analysis (Bailey and Elkan, 1994), searching for 6 motifs for Cbes2777 and 3 motifs for Cbes2780. MAST was used for motif searches with default settings (Bailey and Elkan, 1994). CodonO software (Angellotti et al., 2007) was used with default settings on genome files uploaded from NCBI.

## 3 Results and discussion

### 3.1 The XerD protein encoded by the *C. bescii* native plasmid pBAS2 has homology to known thermophilic proteins but the replication initiation protein, RepL, does not

**Fig. 1:**
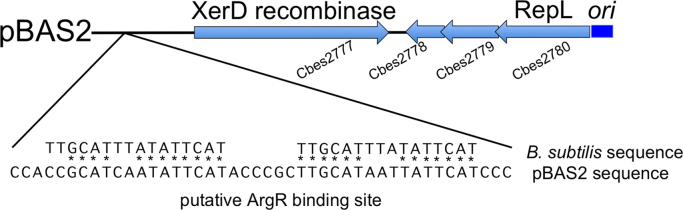
The annotated sequence of *Caldicellulosiruptor bescii* native plasmid pBAS2. Open reading frame numbers are shown below their respective genes, with predicated annotations above the genes. *ori*, conserved putative plasmid replication origin. *XerD recombinase* , site-specific tyrosine recombinase XerD. *RepL*, replication initiation protein. Magnified is the sequence identified by a MAST (Bailey and Elkan, 1994) motif search for the ArgR binding site. The ArgR binding site from *B. subtilis* (Makarova et al., 2001) was used as a query motif. The putative site exists as an approximate direct repeat.

As noted previously (Clausen et al., 2004), the protein sequences encoded by the pBAS2 open reading frames Cbes2777 and Cbes2780 (Fig. 1) are homologous to Xer-like recombinases and the RepL family of replication initiation proteins, respectively. Open reading frames Cbes2778 and Cbes2779 are short ORFs that would encode proteins of 109 aa and 73 aa respectively and are annotated as hypothetical proteins of unknown function (Tables S2 and S3).

**Fig. 2:**
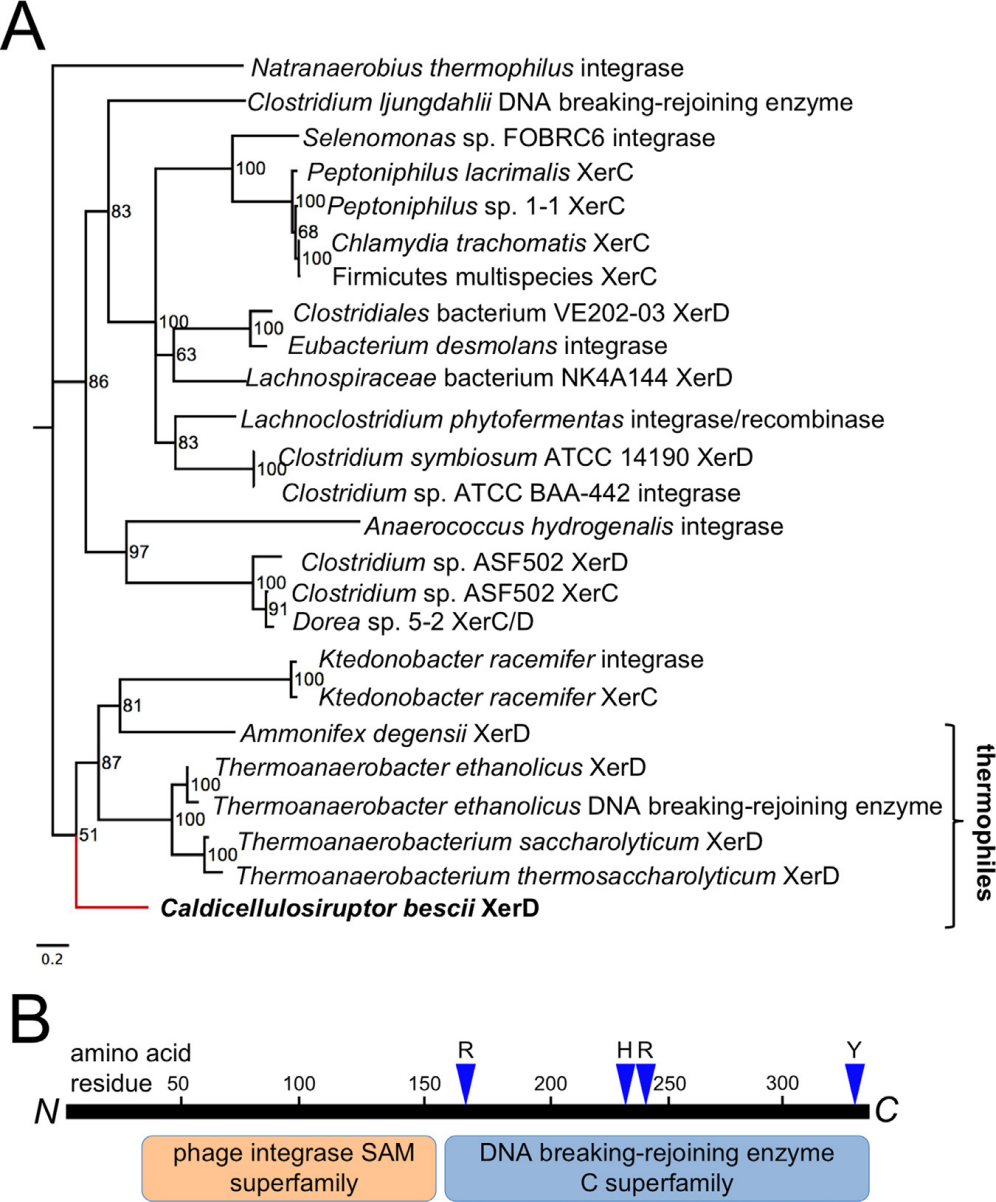
Cbes2777 has thermophilic homologs and a conserved Xer-like catalytic domain. (A) Maximum likelihood tree of Xer-like homologs of Cbes2777. Cbes2777 resides in a cluster with predominantly thermophilic organisms (*T*_opt_≥60 °C), indicated by the bracket. Posterior probabilities determined by MRBAYES are shown at the nodes of the tree. The XerD homolog from *Caldicellulosiruptor bescii* is indicated by a red branch. The scale bar indicates the distance for 0.2 amino acid substitutions per site. (B) Protein domains of the Cbes2777 XerD recombinase. R164, H243, R246, and Y328 appear to constitute the RHRY conserved catalytic residues (Subramanya et al., 1997), indicated above the C-terminal catalytic domain. (For interpretation of the references to color in this figure legend, the reader is referred to the web version of this article.)

Xer recombinases are known to resolve multimers of both chromosomes and plasmids that form during replication (Colloms, 2013, Subramanya et al., 1997). To better understand the replication machinery of the pBAS2 origin, we performed phylogenetic analysis of Cbes2777 and Cbes2780 with more recently acquired sequence data (Geer et al., 2010, Kanehisa and Goto, 2000, Kanehisa et al., 2014). That analysis revealed that Cbes2777 is homologous to XerC and XerD proteins in other Firmicutes, and it weakly clusters with a group of proteins from the thermophilic, anaerobic genera *Thermoanaerobacter* and *Thermoanaerobacterium* (Fig. 2A). This is not surprising, as *Caldicellulosiruptor* is known to be closely related to these genera (Rainey et al., 1993). Motif analysis revealed that all of these Xer-like recombinases contain a phage integrase-like domain and a DNA breaking-rejoining domain (Fig. 2B). The members of the cluster containing Cbes2777 have strong motif signatures for each of these protein domains (Bailey and Elkan, 1994, Marchler-Bauer et al., 2015), in particular the RHRY conserved catalytic active site (Subramanya et al., 1997) (Fig. 2B).

Xer recombination sites for multimer resolution like *cer* on ColE1 and *psi* on pSC101 require extra sequences for the binding of accessory proteins (Cornet et al., 1994, Summers and Sherratt, 1988). One such protein for *cer* is the arginine repressor ArgR (Stirling et al., 1988), which binds to specific sites on the plasmid to orient the recombination sites during multimer resolution. We were unable to identify canonical XerC- and XerD-binding sites (Blake et al., 1997) in the pBAS2 sequence, but a 1.4 kb region of non-coding sequence on plasmid pBAS2 contains predicted ArgR binding sites (Fig. 1) existing as an approximate direct repeat for regulatory protein binding. This site has striking sequence similarity to known ArgR binding sites, particularly those from the *roc* and *car* operons from *B*. *subtilis* (Makarova et al., 2001). Thus, one possibility is that this sequence behaves like *cer* and *psi* for the resolution of plasmid multimers using the XerD recombinase encoded *in cis* by plasmid pBAS2. This would require that the host chromosome encodes an *argR* gene which is, in fact, present in both *C. bescii* and *C. hydrothermalis*, where pBAS2 has previously been shown to replicate. Further, it is present in *C. thermocellum* and other thermophiles including *T*. *saccharolyticum and T*. *ethanolicus* (Marchler-Bauer et al., 2015), suggesting that pBAS2 may have a broader host range than the *Caldicellulosiruptor* genus.

**Fig. 3:**
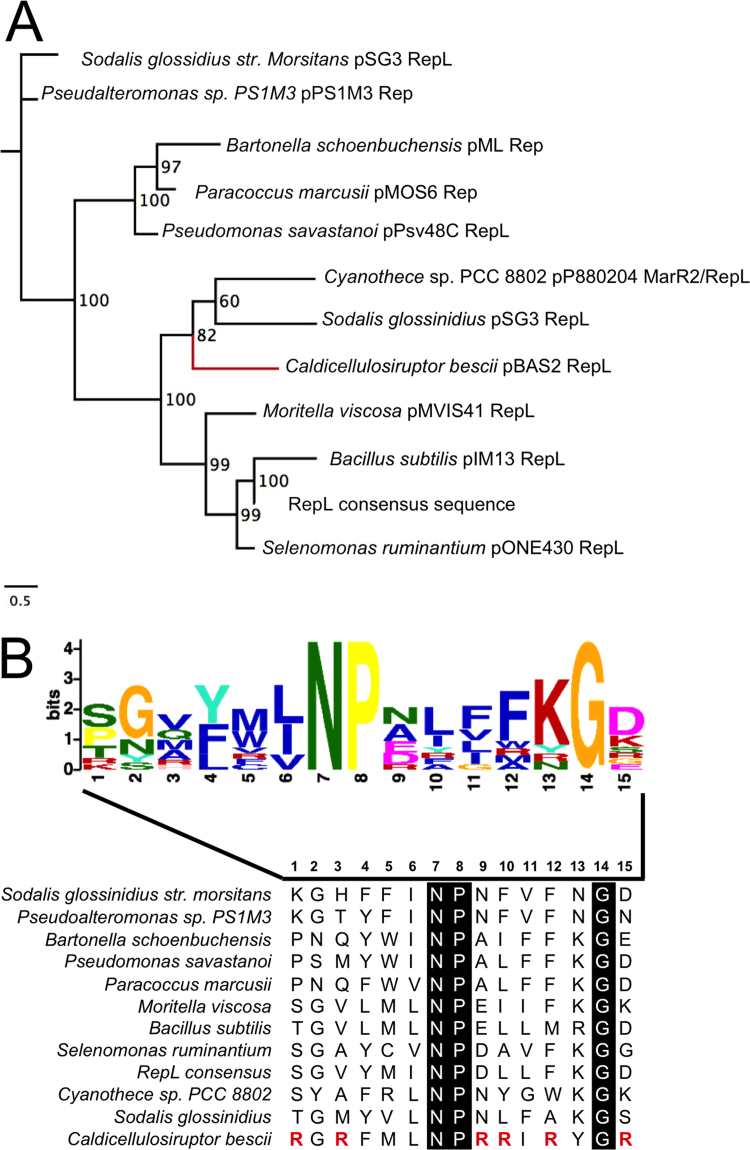
The Cbes2780 RepL protein is unique, but exhibits a conserved motif. (A) Maximum likelihood tree of plasmid-encoded RepL-like homologs to Cbes2780. Plasmid replication proteins are listed with the plasmids that encode them. The RepL consensus sequence is from Sprincova et al. (2005). The scale bar indicates the distance for 0.5 amino acid substitutions per site. (B) The consensus motif of the conserved RepL helix-turn-helix domain generated by MEME (Bailey and Elkan, 1994). In the multiple sequence alignment of 12 plasmid-encoded rep proteins, the conserved N, P and G residues are indicated in black with white font. Sites where the *Caldicellulosiruptor bescii* sequence contains arginine residues that are absent in all other sequences are in red in the multiple sequence alignment.

In contrast to Cbes2777, homology searches for Cbes2780 revealed a limited number of homologs with relatively low sequence identity (≤30%). There were no plasmid-encoded thermophilic homologs, and very few Firmicute homologs (Fig. 3A). Cbes2780 clusters with RepL proteins from diverse bacterial phyla including Proteobacteria, Cyanobacteria, and Firmicutes (Fig. 3A and S1). Among all the replication protein homologs there is a single strong consensus sequence ΦNPX_5_G in a helix-turn-helix DNA binding domain (Fig. 3B), although the *C. bescii* protein has many more arginine residues at this site. It is possible that these residues play a role in thermostability, as arginine residues have been associated with protein thermostability, particularly by increasing hydrophilicity when they replace lysine residues (Barton, 2005, Nadir et al., 1992), and when they exist in clusters (Phillips et al., 2013). These findings, in light of the lack of thermophilic homologs and the fact that Cbes2780 resides on a plasmid, suggest that this protein might have been co-opted by *C. bescii* from a distantly-related mesophilic organism, or alternatively from a currently undiscovered thermophilic organism. Importantly, unique Rep proteins provide for unique incompatibility groups (Shintani et al., 2015) making these plasmids potentially compatible with other known replicons.

It is noteworthy that *Cyanothece* sp. PCC 8802 MarR2/RepL and *Sodalis glossinidius* RepL are encoded on plasmids that encode Xer-like recombinases, reminiscent of the arrangement of RepL and XerD on pBAS2. In fact, the use of portions of pBAS2 to construct other vectors revealed that the XerD recombinase is required for plasmid replication in *C. bescii* (Chung et al., 2013). The organization of pBAS2 may contribute to plasmid promiscuity with both a replication initiation protein and a multimer-resolving recombinase on the same plasmid.

### 3.2 A thermostable replicon from the *C. bescii* plasmid pBAS2 transforms *C. thermocellum* at 60 °C

**Fig. 4:**
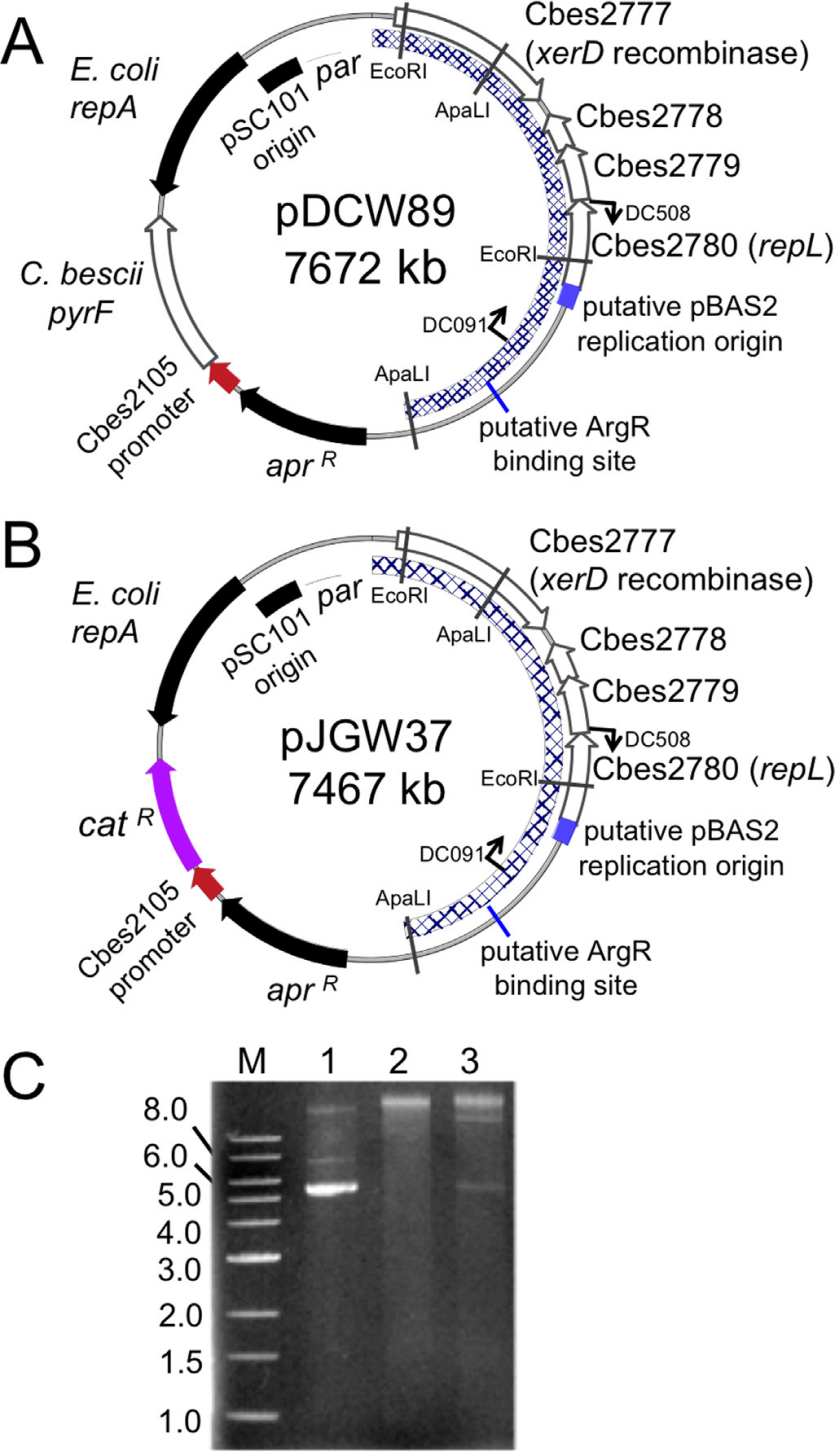
Maps of shuttle vectors transformed into *Clostridium thermocellum*. (A) pDCW89 constructed with the *C. bescii pyrF* gene driven by the *C. bescii* Cbes2105 ribosomal protein S30A promoter. The hatched region was derived from *C. bescii* native plasmid pBAS2. *Apr^R^*, apramycin resistance casette; *repA*, replication initiator for *E. coli* pSC101 replication origin; *par*, partitioning locus for *E. coli*. Primers for PCR verification of transformation and restriction sites for structural verification are shown on the plasmid map. (B) pJGW37 is identical to pDCW89, but with the chloramphenicol acetyltransferase gene (*cat*) as the selectable marker. (C) Plasmid DNA can be visualized in total DNA directly purified from *C. thermocellum*. 1: 500 ng pJGW37 purified from *E. coli*. 2: 1.3 μg total DNA purified from *C. thermocellum Δhpt*. 3: 1.3 μg total DNA purified from *C. thermocellum Δhpt* containing pJGW37.

Two pBAS2-derived plasmids were used for testing transformation of *C. thermocellum*. Plasmid pDCW89 (Fig. 4A) was previously constructed from the native *C. bescii* plasmid pBAS2 (Fig. 1) (Clausen et al., 2004) for use as an *E. coli*/*Caldicellulosiruptor* shuttle vector (Chung et al., 2013). It contains the pBAS2 origin of replication, the *E. coli* plasmid pSC101 origin of replication, an apramycin resistance cassette for selection in *E. coli,* and the *C. bescii pyrF* wild type allele used to select uracil prototrophy in strains containing a deletion of the *pyrF* gene. Previously, a *C. thermocellum pyrF* deletion was constructed (Tripathi et al., 2010), allowing transformation of pDCW89 to be tested (see below) using uracil prototrophy as the selection. Because thiamphenicol selection is also commonly used for *C. thermocellum* transformations, we constructed plasmid pJGW37 (Fig. 4B). This plasmid was based on pDCW89 (Fig. 4A) but with the *pyrF* gene replaced with a chloramphenicol acetyltransferase (*cat*) gene for selection of transformants in *C. thermocellum* strains that are wild type at the *pyrF* locus.

Using a method developed for *C. bescii* (Chung et al., 2012), pDCW89 was electrotransformed into a *C. thermocellum* strain containing a deletion of the *pyrF* gene (Tripathi et al., 2010). As with the *C. bescii pyrF* deletion, this strain is a uracil auxotroph resistant to 5-fluoroorotic acid (5-FOA) (Table 1), allowing selection and counter-selection of the *pyrF* wild type allele. Transformants of pDCW89 were successfully selected for uracil prototrophy in defined liquid medium at 60 °C. Further, plasmid pJGW37 was successfully transformed into *C. thermocellum* containing a deletion of the hypoxanthine phosphoribosyl transferase (*hpt*) (Argyros et al., 2011) selecting thiamphenicol resistance, again at 60 °C. Hpt forms nucleotide monophosphates from purines, and can lead to the incorporation of toxic purine analogs such as 8-azahypoxanthine (AZH) into DNA and RNA (Stout and Caskey, 1985). The *Δhpt* strain is resistant to AZH allowing selection of transformants that are thiamphenicol resistant and subsequent counter selection for AZH resistance.

**Table 1:** Strains and plasmids used in this study.

Strain or plasmid	Genotype/phenotype	Source
*C. thermocellum*		
LL1005	*C. thermocellum* DSM 1313 Δ*pyrF* (*ura*^*−*^/5−FOA^R^)	Tripathi et al. (2010)
M1354	*C. thermocellum* DSM 1313 Δ*hpt*	Argyros et al. (2011)
JWCT02	*C. thermocellum ΔpyrF*+pDCW89 (*ura*^*+*^/5-FOA^S^)	This work
JWCT03	*C. thermocellum ΔpyrF*+pDCW196 (*ura*^*+*^/5-FOA^S^/Tm^R^)	This work
JWCT04	*C. thermocellum Δhpt*+pDCW196 (*ura*^*+*^/5-FOA^S^/Tm^R^)	This work
JWCT05	*C. thermocellum Δhpt*+pJGW37 (Tm^R^)	This work
		
*E. coli*		
JW421	BL21 containing pJGW37 (Apramycin^R^)	This work
JW422	BL21 containing pDCW89 (Apramycin^R^)	This work
		
Plasmids		
pDCW89	*E. coli*/*C. bescii* shuttle vector (P_Cbes2105_-*pyrF*)	Chung et al. (2012)
pDCW196	*E. coli*/*C. bescii* shuttle vector (P_Cbes2105_*-pyrF*/P_gapDH_-*cat*)	This work
pJGW37	*E. coli*/*C. bescii* shuttle vector (P_Cbes2105_-*cat*)	This work

Total DNA isolated from *C. thermocellum* transformants containing pDCW89 or *C. thermocellum* transformants containing pJGW37 was used to back-transform *E. coli*. Two different restriction endonuclease digests performed on plasmid DNA purified from nine independent *E. coli* colonies (3 shown) resulted in identical banding patterns relative to the original plasmid (Fig. S2). This proves that the plasmids were successfully transformed into *C. thermocellum* and suggests that there was no major structural instability of the plasmid during transformation and replication in *C. thermocellum* and back-transformation into *E. coli.* Plasmid pJGW37 was also purified directly from *C. thermocellum* and could be visualized on an agarose gel of total DNA (Fig. 4C).

### 3.3 Plasmid stability, copy number and transformation efficiency

To assess transformation efficiency of the pBAS2 replicon, cells were transformed with pJGW37 and plated after a 6 h recovery period in rich recovery medium onto plates with thiamphenicol at 60 °C. Transformation efficiency of pJGW37 was determined to be 3242±575 colony-forming units per µg plasmid DNA (CFU/µg), demonstrating that the pBAS2 origin of replication can efficiently transform *C. thermocellum* at 60 °C. This is in contrast to plasmids containing the pNW33N replicon, where the temperature limit for transformation is~51 °C (Olson and Lynd, 2012a).

**Fig. 5:**
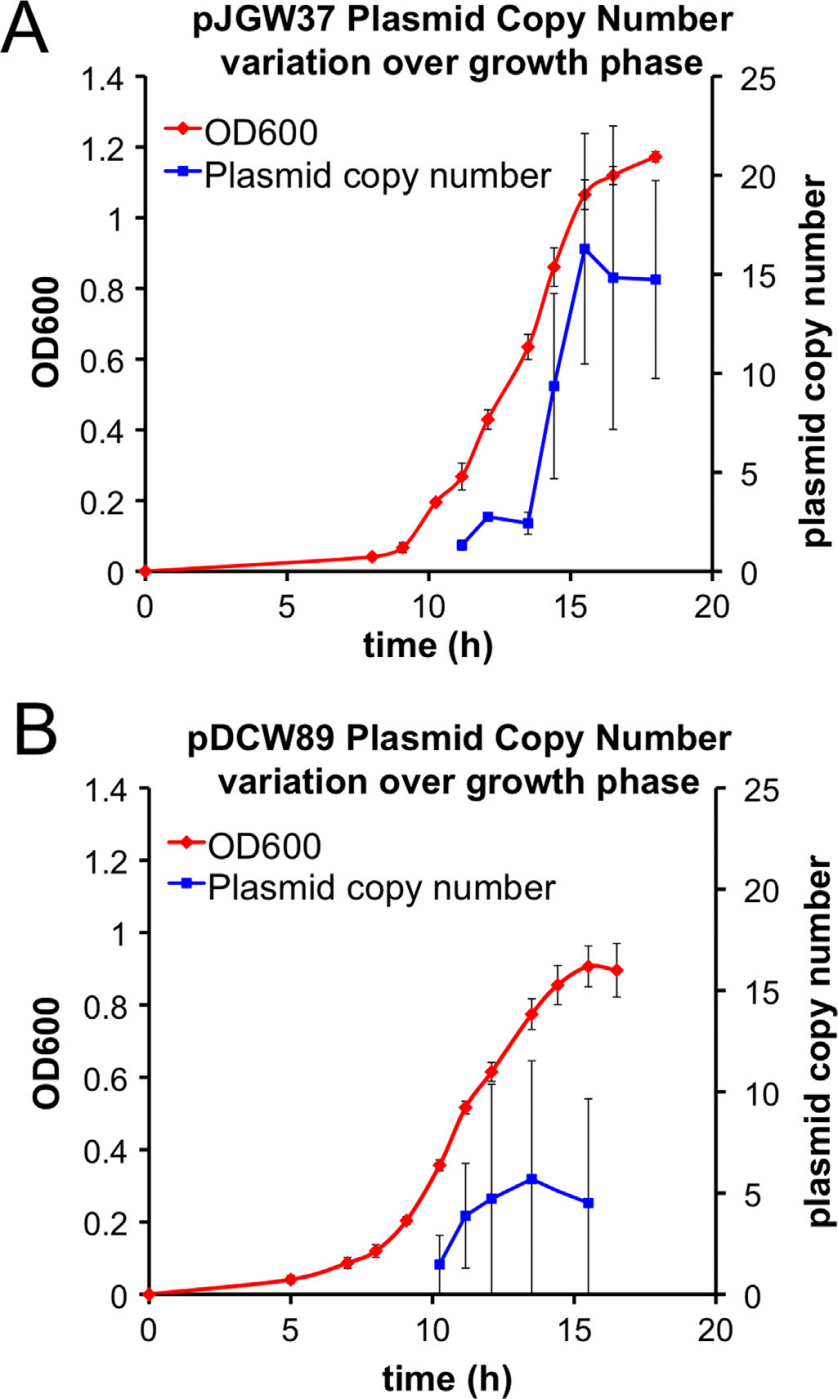
Plasmid copy number is dependent on growth phase. Plasmid pJGW37 (A) was maintained with thiamphenicol resistance, while pDCW89 (B) was maintained with uracil auxotrophy. Growth of triplicate cultures at 60 °C determined by OD_600_ is portrayed in red. 2% (v/v) samples for plasmid copy number analysis were taken from the standing cultures. Plasmid copy number (PCN), indicated by blue squares, represents the copies of plasmid per chromosome as measured by qPCR. (For interpretation of the references to color in this figure legend, the reader is referred to the web version of this article.)

To assess plasmid stability and copy number, individual *C. thermocellum* transformants were passaged in both selective and non-selective media five times, and the copy number was measured using quantitative polymerase chain reaction (qPCR), as described by Lee et al. (2006). The plasmid copy number (PCN) varied depending on the selection method and the growth phase but was highest in late exponential phase for pJGW37 at 10–20 copies per chromosome (Fig. 5A) and the PCN for pDCW89 varied from 2 to 10 during both exponential and stationary phases (Fig. 5B). The reason for the differences in copy number with growth phase and growth rate may reflect the fact that copy number is an average across the population. After one passage without selection, the copy number is below 1, suggesting that either 10% of the population lost the plasmid, or more likely, most contain the plasmid in multiple copies, and some have lost it. Without selection, both plasmids decreased in average copy number with successive serial passages and were lost after five passages (Table 2).

**Table 2:** Determination of plasmid copy number.

	Passages with selection		Passages without selection		
Plasmid	**1**	**5**	**1**	**2**	**5**
pDCW89	3.0±0.4	9.2±0.9	1.7±1.1	0.1±0.04	0
pJGW37	6.7±0.6	7.5±1.7	0.9±0.1	0.1±0.05	0


Plasmid copy number was determined at mid-log phase after the indicated number of passages. Maintenance experiments were performed in biological duplicate. Uracil prototrophy was used to select for pDCW89. Resistance to thiamphenicol was used to select for pJGW37.

### 3.4 Heterologous proteins and promoters from *C. bescii* function in *C. thermocellum*

Plasmid pDCW89 contains the *C. bescii pyrF* wild type allele and this gene functions to complement the *pyrF* deletion in *C. thermocellum*. The PyrF proteins have 45% amino acid sequence identity. The G+C content of the *C. bescii pyrF* gene is 35.7%, and that of the *C. thermocellum pyrF* gene is 39.9%. In fact, CodonO software (Angellotti et al., 2007) indicates that codon usage bias between the entire *C. bescii* and *C. thermocellum* genomes is not statistically significant with a *p* value of >0.33. Both pDCW89 and pJGW37 rely on a *C. bescii* ribosomal protein promoter (Chung et al., 2013) to direct transcription of the selectable marker gene (Fig. 1), the *pyrF* gene in pDCW89 and the *cat* gene in pJGW37. While we have no direct evidence for the efficiency of these promoters at this time, they clearly function at a level sufficient to allow selection. No promoter from *C. bescii* has been characterized experimentally so the transcription start site and RNA polymerase binding site for this promoter is unknown, but the sequence does contain a prototypical RNA polymerase binding site sequence for growth phase dependent transcription in Gram-positive bacteria.

## 4 Conclusions

While many plasmid origins of replication are derived from mesophilic sources, genetic tools for thermophiles must function at elevated temperatures. The use of thermophilic sources offers a solution to this issue, and we have demonstrated that plasmid origin pBAS2 from thermophile *C. bescii* can replicate in *C. thermocellum* at its optimal growth temperature of 60 °C. This expansion of the genetic tools available for *C. thermocellum* will facilitate more rapid genetic engineering toward the goal of developing an organism for the efficient conversion of lignocellulosic biomass to fuels and chemicals. Further, the demonstration of stable, autonomous replication of the *C. bescii* pBAS2 replicon in *C. thermocellum* suggests that this replicon might serve as a new tool for plasmid-based expression in other thermophiles, as well.

## Conflict of interest

LL has an equity interest in Enchi Corporation, a biofuel start-up company with a financial interest in *C. thermocellum*.

## Footnotes

Appendix A Supplementary data associated with this article can be found in the online version at doi:10.1016/j.meteno.2016.01.004.

## References

[bib1] Akinosho H., Yee K., Close D., Ragauskus A. (2014). The emergence of *Clostridium thermocellum* as a high utility candidate for consolidated bioprocessing applications. Front. Chem..

[bib2] Angellotti M.C., Bhuiyan S.B., Chen G., Wan X.F. (2007). CodonO: codon usage bias analysis within and across genomes. Nucleic Acids Res..

[bib3] Argyros D.A., Tripathi S.A., Barrett T.F., Rogers S.R., Feinberg L.F., Olson D.G., Caiazza N.C. (2011). High ethanol titers from cellulose by using metabolically engineered thermophilic, anaerobic microbes. Appl. Environ. Microbiol..

[bib4] Bailey T.L., Elkan C. (1994). Fitting a mixture model by expectation maximization to discover motifs in biopolymers. Proc. Int. Conf. Intell. Syst. Mol. Biol..

[bib5] Barton L.L. (2005). Thermostability of Proteins. Structural and Functional Relationships in Prokaryotes.

[bib6] Bayer E.A., Shoham Y., Lamed R., Rosenberg E. (2013). Lignocellulose-decomposing bacteria and their enzyme systems. The Prokaryotes – Prokaryotic Physiology and Biochemistry.

[bib7] Biswas R., Zheng T., Olson D.G., Lynd L.R., Guss A.M. (2015). Elimination of hydrogenase active site assembly blocks H2 production and increases ethanol yield in *Clostridium thermocellum*. Biotechnol. Biofuels.

[bib8] Blake J.A., Ganguly N., Sherratt D.J. (1997). DNA sequence of recombinase-binding sites can determine Xer site-specific recombination outcome. Mol. Microbiol..

[bib9] Chung D., Cha M., Farkas J., Westpheling J. (2013). Construction of a stable replicating shuttle vector for *Caldicellulosiruptor* species: use for extending genetic methodologies to other members of this genus. PLoS One.

[bib10] Chung D., Farkas J., Huddleston J.R., Olivar E., Westpheling J. (2012). Methylation by a unique alpha-class N4-cytosine methyltransferase is required for DNA transformation of *Caldicellulosiruptor bescii* DSM6725. PLoS One.

[bib11] Chung D., Young J., Bomble Y.J., Vander Wall T.A., Groom J., Himmel M.E., Westpheling J. (2015). Homologous expression of the *Caldicellulosiruptor bescii* CelA reveals that the extracellular protein is glycosylated. PLoS One.

[bib12] Clausen A., Mikkelsen M.J., Schroder I., Ahring B.K. (2004). Cloning, sequencing, and sequence analysis of two novel plasmids from the thermophilic anaerobic bacterium *Anaerocellum thermophilum*. Plasmid.

[bib13] Colloms S.D. (2013). The topology of plasmid-monomerizing Xer site-specific recombination. Biochem. Soc. Trans..

[bib14] Cornet F., Mortier I., Patte J., Louarn J.M. (1994). Plasmid pSC101 harbors a recombination site, *psi*, which is able to resolve plasmid multimers and to substitute for the analogous chromosomal *Escherichia coli* site *dif*. J. Bacteriol..

[bib15] Darriba D., Taboada G.L., Doallo R., Posada D. (2011). ProtTest 3: fast selection of best-fit models of protein evolution. Bioinformatics.

[bib16] Demain A.L., Newcomb M., Wu D.J.H. (2005). Cellulase, clostridia, and ethanol. Microbiol. Mol. Biol. Rev..

[bib17] Deng Y., Olson D.G., Zhou J., Herring C.D., Shaw J.A. (2013). Redirecting carbon flux through exogenous pyruvate kinase to achieve high ethanol yields in *Clostridium thermocellum*. Metab. Eng..

[bib18] Dubnau D. (1983). Molecular Cloning in Bacillus subtilis.

[bib19] Ehrlich S.D. (1977). Replication and expression of plasmids from *Staphylococcus aureus* in *Bacillus substilis*. Proc. Natl. Acad. Sci. USA.

[bib20] Garnier T., Cole S.T. (1988). Complete nucleotide sequence and genetic organization of the bacteriocinogenic plasmid, pIP404, from *Clostridium perfringens*. Plasmid.

[bib21] Geer L.Y., Marchler-Bauer A., Geer R.C., Han L., He J., He S., Bryant S.H. (2010). The NCBI BioSystems database. Nucleic Acids Res..

[bib22] Goujon M., McWilliam H., Li W., Valentin F., Squizzato S., Paern J., Lopez R. (2010). A new bioinformatics analysis tools framework at EMBL-EBI. Nucleic Acids Res..

[bib23] Groom J., Chung D., Young J., Westpheling J. (2014). Heterologous complementation of a *pyrF* deletion in *Caldicellulosiruptor hydrothermalis* generates a new host for the analysis of biomass deconstruction. Biotechnol. Biofuels.

[bib24] Guss A.M., Olson D.G., Caiazza N.C., Lynd L.R. (2012). Dcm methylation is detrimental to plasmid transformation in *Clostridium thermocellum*. Biotechnol. Biofuels.

[bib25] Huelsenbeck J.P., Ronquist F. (2001). MRBAYES: bayesian inference of phylogenetic trees. Bioinformatics.

[bib26] Jannière L., Bruand C., Ehrlich S.D. (1990). Structurally stable *Bacillus subtilis* cloning vectors. Gene.

[bib27] Kanehisa M., Goto S. (2000). KEGG: kyoto encyclopedia of genes and genomes. Nucleic Acids Res..

[bib28] Kanehisa M., Goto S., Sato Y., Kawashima M., Furumichi M., Tanabe M. (2014). Data, information, knowledge and principle: back to metabolism in KEGG. Nucleic Acids Res..

[bib29] Khan S.A. (2005). Plasmid rolling-circle replication: highlights of two decades of research. Plasmid.

[bib30] Klapatch T.R., Guerinot M.L., Lynd L.R. (1996). Electrotransformation of *Clostridium thermosaccharolyticum*. J. Ind. Microbiol..

[bib31] Lamed R., Bayer E.A., Allen I.L. (1988). The cellulosome of *Clostridium thermocellum*.

[bib32] Le S.Q., Gascuel O. (2008). An improved general amino acid replacement matrix. Mol. Biol. Evol..

[bib33] Lee C., Ow D., Oh S. (2006). Quantitative real-time polymerase chain reaction for determination of plasmid copy number in bacteria. J. Microbiol. Methods.

[bib34] Lin P.P., Mi L., Morioka A.H., Yoshino K.M., Konishi S., Xu S.C., Liao J.C. (2015). Consolidated bioprocessing of cellulose to isobutanol using *Clostridium thermocellum*. Metab. Eng..

[bib35] Luchansky J.B., Muriana P.M., Klaenhammer T.R. (1988). Application of electroporation for transfer of plasmid DNA to *Lactobacillus*, *Lactococcus*, *Leuconostoc*, *Listeria*, *Pediococcus*, *Bacillus*, *Staphylococcus*, *Enterococcus* and *Propionibacterium*. Mol. Microbiol..

[bib36] Mai V., Lorenz W.W., Wiegel J. (1997). Transformation of *Thermoanaerobacterium sp.* strain JW/SL-YS485 with plasmid pIKM1 conferring kanamycin resistance. FEMS Microbiol. Lett..

[bib37] Makarova K.S., Mironov A.A., Gelfand M.S. (2001). Conservation of the binding site for the arginine repressor in all bacterial lineages. Genome Biol..

[bib38] Marchler-Bauer A., Derbyshire M.K., Gonzales N.R., Lu S., Chitsaz F., Geer L.Y., Bryant S.H. (2015). CDD: NCBI's conserved domain database. Nucleic Acids Res..

[bib39] McBee R.H. (1954). The characteristics of *Clostridium thermocellum*. J. Bacteriol..

[bib40] McNamara P.J., Lindsay J. (2008). Chapter 5: genetic manipulation of *Staphylococcus aureus*. Staphylococcus: Molecular Genetics.

[bib41] Mearls E.B., Olson D.G., Herring C.D., Lynd L.R. (2015). Development of a regulatable plasmid-based gene expression system for *Clostridium thermocellum*. Appl. Microbiol. Biotechnol..

[bib42] Nadir T.M., Annemie Van den B., Ilse Van den B., Patrick B., Yves S., Anne Marie L., Mohamed C. (1992). Arginine residues as stabilizing elements in proteins. Biochemistry.

[bib43] Olson D.G., Lynd L.R. (2012). Chapter 17: Transformation of *Clostridium thermocellum* by electroporation. Methods Enzymol..

[bib44] Olson D.G., Lynd L.R. (2012). Computational design and characterization of a temperature-sensitive plasmid replicon for gram positive thermophiles. J. Biol. Eng..

[bib45] Olson D.G., Maloney M., Lanahan A.A., Hon S., Hauser L.J., Lynd L.R. (2015). Identifying promoters for gene expression in *Clostridium thermocellum*. Metab. Eng. Commun..

[bib46] Papanek B.A., Biswas R., Rydzak T., Guss A.M. (2015). Elimination of metabolic pathways to all traditional fermentation products increases ethanol yields in *Clostridium thermocellum*. Metab. Eng..

[bib47] Peng H., Fu B., Mao Z., Shao W. (2006). Electrotransformation of *Thermoanaerobacter ethanolicus JW200*. Biotechnol. Lett..

[bib48] Phillips L.G., Whitehead D.M., Kinsella J.E. (2013). Ch. 2 Section 6: Hydrophobicity and Protein Stability. Structure-Function Properties of Food Proteins.

[bib49] Pritchett M.A., Zhang J.K., Metcalf W.W. (2004). Development of a markerless genetic exchange method for *Methanosarcina acetivorans C2A* and its use in construction of new genetic tools for methanogenic archaea. Appl. Environ. Microbiol..

[bib50] Projan S.J., Monod M., Narayanan C.S., Dubnau D. (1987). Replication properties of pIM13, a naturally occurring plasmid found in *Bacillus subtilis*, and of its close relative pE5, a plasmid native to *Staphylococcus aureus*. J. Bacteriol..

[bib51] Rainey F.A., Ward N.L., Morgan H.W., Toalster R., Stackebrandt E. (1993). Phylogenetic analysis of anaerobic thermophilic bacteria: aid for their reclassification. J. Bacteriol..

[bib52] Serwold-Davis T.M., Groman N., Rabin M. (1987). Transformation of Corynebacterium diphtheriae, Corynebacterium ulcerans, Corynebacterium glutamicum, and Escherichia coli with the C. diphtheriae plasmid pNG2. Proc. Natl. Acad. Sci. USA.

[bib53] Shintani M., Sanchez Z.K., Kimbara K. (2015). Genomics of microbial plasmids: classification and identification based on replication and transfer systems and host taxonomy. Front. Microbiol..

[bib54] Sievers F., Wilm A., Dineen D., Gibson T.J., Karplus K., Li W., Higgins D.G. (2011). Fast, scalable generation of high-quality protein multiple sequence alignments using Clustal Omega. Mol. Syst. Biol..

[bib55] Sprincova A., Javorsky P., Pristas P. (2005). pSRD191, a new member of RepL replicating plasmid family from *Selenomonas ruminantium*. Plasmid.

[bib56] Stirling C.J., Szatmari G., Stewart G., Smith M.C., Sherratt D.J. (1988). The arginine repressor is essential for plasmid-stabilizing site-specific recombination at the ColE1 *cer* locus. EMBO J..

[bib57] Stout J.T., Caskey C.T. (1985). HPRT: gene structure, expression, and mutation. Annu. Rev. Genet..

[bib58] Subramanya H.S., Arciszewska L.K., Baker R.A., Bird L.E., Sherratt D.J., Wigley D.B. (1997). Crystal structure of the site‐specific recombinase, XerD. EMBO J..

[bib59] Summers D.K., Sherratt D.J. (1988). Resolution of ColE1 dimers requires a DNA sequence implicated in the three-dimensional organization of the *cer* site. EMBO J..

[bib60] Tripathi S.A., Olson D.G., Argyros D.A., Miller B.B., Barrett T.F., Murphy D.M., Caiazza N.C. (2010). Development of *pyrF*-based genetic system for targeted gene deletion in *Clostridium thermocellum* and creation of a *pta* mutant. Appl. Environ. Microbiol..

[bib61] Tyurin M.V., Desai S.G., Lynd L.R. (2004). Electrotransformation of *Clostridium thermocellum*. Appl. Environ. Microbiol..

[bib62] Whelan S., Goldman N. (2001). A general empirical model of protein evolution derived from multiple protein families using a maximum-likelihood approach. Mol. Biol. Evol..

[bib63] Yang S.J., Kataeva I., Hamilton-Brehm S.D., Engle N.L., Tschaplinski T.J., Doeppke C., Adams M.W. (2009). Efficient degradation of lignocellulosic plant biomass, without pretreatment, by the thermophilic anaerobe “*Anaerocellum thermophilum*” DSM 6725. Appl. Environ. Microbiol..

