# Supplementary material for: Promiscuous plasmid replication in thermophiles: Use of a novel hyperthermophilic replicon for genetic manipulation of Clostridium thermocellum at its optimum growth temperature
Source: Metab Eng Commun. 2016 Jan 29;3:30–8. doi: 10.1016/j.meteno.2016.01.004 (PMC5779722; doi:10.1016/j.meteno.2016.01.004)
Supplement: Supplementary file 1 — Supplementary material [file mmc1.docx]

| **Supplementary Table S1. Primers used in this study** | |
| --- | --- |
| DC091 | ATCTTCATGATTTTCCCAGGA |
| DC508 | AGA GGTACC AGTTCCTGCTTTGTTAACATTCCTTG |
| X013 | AGA GGATCC TTACTTCCTGTCTCGCAACGC |
| X014 | AGA CATATG GACAGTTTTCCCTTTGATATGT |
| X015 | AGA GGATCC TGCAATAATTACTGTATCTCTCTGGCA |
| X016 | AGA CATATG CTTCAAACTTCCCAAAGGCGAGCCCT |
| JG024 | AGATCA TCTAGA GACCATCCTTTCTATGTAGAAA |
| JG099 | AAGA TCTAGA ATGAACTTTAATAAAATTGATTTAGACAATTGGAA |
| Q1 | TGGGAAAGCCGTCCATAATC |
| Q2 | TCTCCCGCTCTTCTCTCTTT |
| Q3 | GTGCGTCTACAGGACCTTATTT |
| Q4 | GGCAAGATTCTACAGGCAAGA |
| CTQ1 | CCAAACCTCCTTCCCGATATAC |
| CTQ2 | CTCTCAGCTCCTCATCCTCTAT |
| CTQ3 | GGAACCGGAGTGAATGTCATAG |
| CTQ4 | CTGGGAATTGTAGCCCGAATAA |

| **Supplementary Table S2. Homologous proteins to Cbes2778** | | | |
| --- | --- | --- | --- |
| **Organism** | **Annotation** | **Query cover** | **% identity** |
| *Caldicellulosiruptor saccharolyticus* | Hypothetical protein | 94% | 43% |
| *Caldicellulosiruptor sp. Rt8.B8* | Hypothetical protein | 75% | 42% |
| *Caldicellulosiruptor kronotskyensis* | Hypothetical protein | 90% | 35% |
| *Spiroplasma apis* | FeS assembly protein | 76% | 32% |
| *Lumbricus terrestris* | [extracellular hemoglobin linker chain (N-terminal)](http://blast.ncbi.nlm.nih.gov/Blast.cgi#alnHdr_1332729) | 69% | 33% |
| *Lumbricus terrestris* | [Hemoglobin complex, chain 2](http://blast.ncbi.nlm.nih.gov/Blast.cgi#alnHdr_636665998) | 72% | 34% |
| *Lumbricus terrestris* | Extracellular hemoglobin linker L3 subunit precursor | 72% | 34% |
| *Lumbricus terrestris* | Chain O, Lumbricus Erythrocruorin | 72% | 34% |
| *Acidithiobacillus thiooxidans* | hypothetical protein | 47% | 43% |
| *Caldicellulosiruptor saccharolyticus* | hypothetical protein | 42% | 45% |
| *Acidithiobacillus thiooxidans* | hypothetical protein | 47% | 43% |
| *Kluyvera ascorbata* | hypothetical protein | 61% | 27% |

BLAST analysis identified five hypothetical protein potential homologs to Cbes2778 in the *Caldicellulosiruptor* genus with relatively high query coverage and sequence identity. Multiple additional similar proteins were identified, mostly from eukaryotes.

| **Supplementary Table S3. Homologous proteins to Cbes2779** | | | |
| --- | --- | --- | --- |
| **Organism** | **Annotation** | **Query cover** | **% identity** |
| *Eubacterium cellulolosolves* | Transketolase | 48% | 33% |
| *Buchnera aphidocola* | GTPase EngA | 84% | 32% |
| *Prolixibacter bellariivorans* | Hypothetical protein | 54% | 31% |
| *Stanieria cyanosphaera* | Ferritin Dps family protein | 41% | 33% |

Cbes2779 has no homologs in *Caldicellulosiruptor* and shows only weak sequence similarity to any other bacterial proteins.


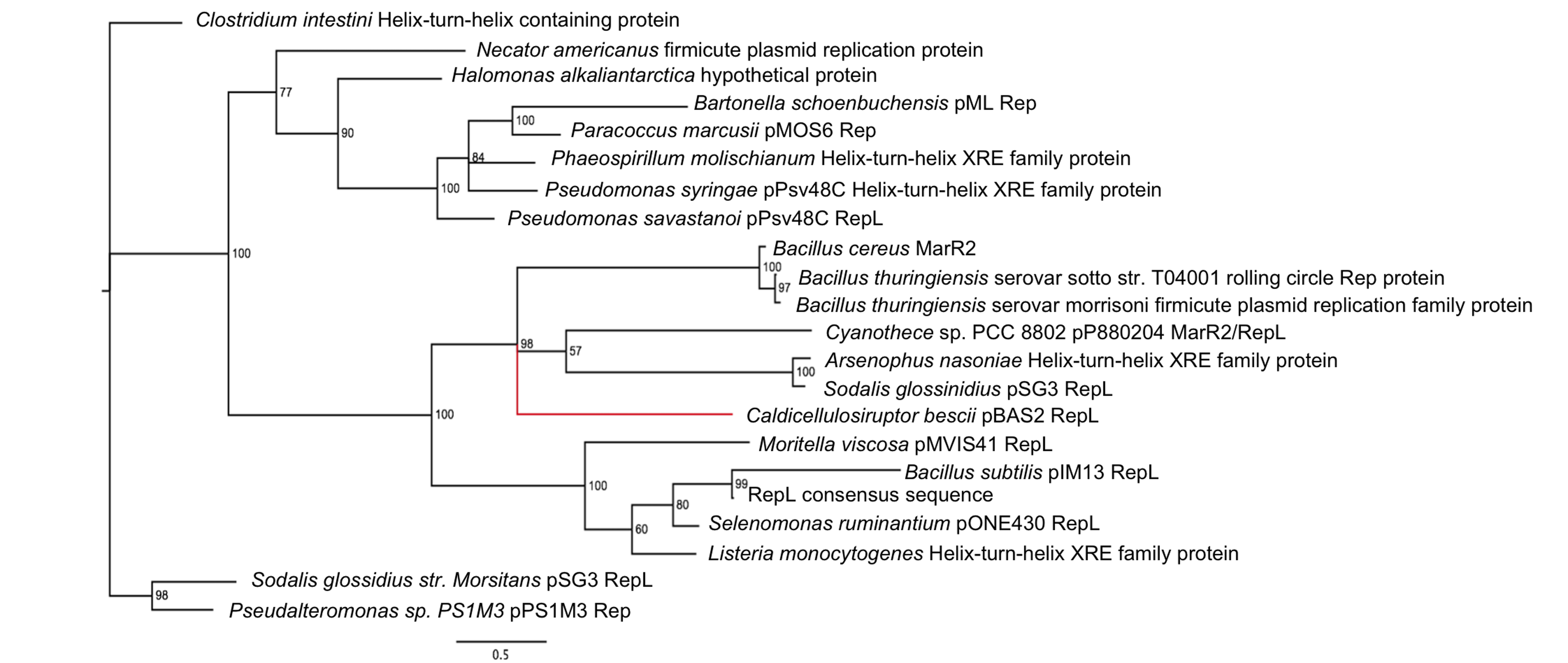


**Supplementary Figure S1.** **Maximum likelihood tree of full list of RepL-like homologs to Cbes2780**. The tree contains both plasmid-encoded and chromosome-encoded homologs to Cbes2780. Plasmid replication proteins are listed with the plasmids that encode them. The RepL consensus sequence is from Sprincova *et al* (Sprincova et al., 2005). The scale bar indicates the distance for 0.5 amino acid substitutions per site.


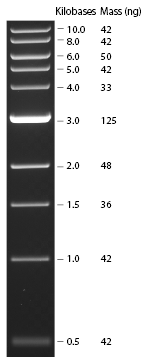


A)

M 1 2 3 4 5 6 7 8


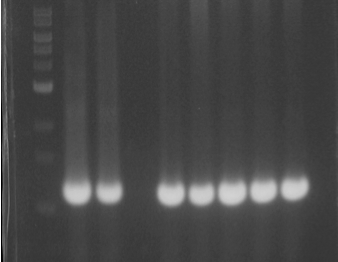


C)

M 1 2 3 4 5 6 7 8 M


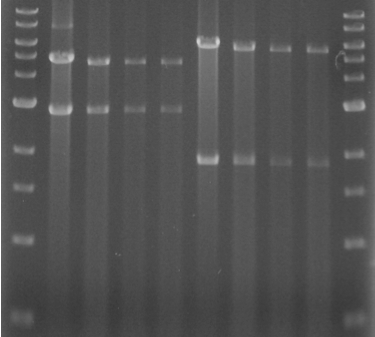

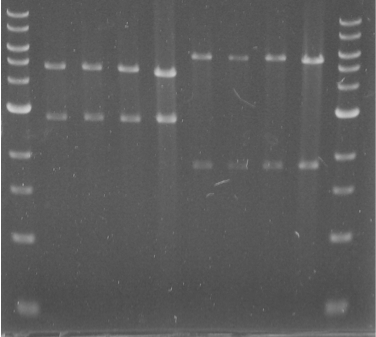


M 1 2 3 4 5 6 7 8 M

B)

**Supplementary Figure S2. Plasmids with the pBAS2 replication origin are structurally stable in *C. thermocellum* 1313. A)** PCR verification of the pDCW89 plasmid in *C. thermocellum* Δ*pyrF* transformants using plasmid-specific primers DC091/DC508 (See Fig. 1). **1:** plasmid pDCW89; **2:** plasmid pJGW37; **3:** negative control *C. thermocellum* Δ*pyrF* DNA; **4-6:** total DNA from uracil prototrophic pDCW89 transformants; **7-8**: total DNA from thiamphenicol resistant pJGW37 transformants. **B)** Back-transformation of pJGW37 isolated from *C. thermocellum* into *E. coli*, followed by restriction digest*.* **1,5:** pJGW37 purified from *E. coli*; **2-4, 6-8:** plasmid isolated from individual *E. coli* colonies back-transformed with *C. thermocellum* DNA; **1-4:** Cut with EcoRI; **5-8:** Cut with ApaLI. **C)** Back-transformation of pDCW89 isolated from *C. thermocellum* into *E. coli*, followed by restriction digest*.* **1,5:** pDCW89 purified from *E. coli*; **2-4, 6-8:** plasmid isolated from individual *E.coli* colonies back-transformed with *C. thermocellum* DNA; **1-4:** Cut with EcoRI; **5-8:** Cut with ApaLI. DNA ladder is the 1 kb Ladder from NEB.
